# Molecular elevation of insulin receptor signaling improves memory recall in aged Fischer 344 rats

**DOI:** 10.1111/acel.13220

**Published:** 2020-08-27

**Authors:** Hilaree N. Frazier, Katie L. Anderson, Adam O. Ghoweri, Ruei-Lung Lin, Tara R. Hawkinson, Gabriel J. Popa, Pradoldej Sompol, Michael D. Mendenhall, Christopher M. Norris, Olivier Thibault

**Affiliations:** ^1^ Department of Pharmacology and Nutritional Sciences Lexington Kentucky USA; ^2^ Department of Molecular and Cellular Biochemistry Lexington Kentucky USA; ^3^ Sanders-Brown Center on Aging University of Kentucky Lexington Kentucky USA

**Keywords:** brain, gait, genetic engineering, hippocampus, insulin resistance, spatial memory

## Abstract

As demonstrated by increased hippocampal insulin receptor density following learning in animal models and decreased insulin signaling, receptor density, and memory decline in aging and Alzheimer's diseases, numerous studies have emphasized the importance of insulin in learning and memory processes. This has been further supported by work showing that intranasal delivery of insulin can enhance insulin receptor signaling, alter cerebral blood flow, and improve memory recall. Additionally, inhibition of insulin receptor function or expression using molecular techniques has been associated with reduced learning. Here, we sought a different approach to increase insulin receptor activity without the need for administering the ligand. A constitutively active, modified human insulin receptor (IRβ) was delivered to the hippocampus of young (2 months) and aged (18 months) male Fischer 344 rats in vivo. The impact of increasing hippocampal insulin receptor expression was investigated using several outcome measures, including Morris water maze and ambulatory gait performance, immunofluorescence, immunohistochemistry, and Western immunoblotting. In aged animals, the IRβ construct was associated with enhanced performance on the Morris water maze task, suggesting that this receptor was able to improve memory recall. Additionally, in both age‐groups, a reduced stride length was noted in IRβ‐treated animals along with elevated hippocampal insulin receptor levels. These results provide new insights into the potential impact of increasing neuronal insulin signaling in the hippocampus of aged animals and support the efficacy of molecularly elevating insulin receptor activity in vivo in the absence of the ligand to directly study this process.

## Graphical abstract

To assess the impact of increasing insulin signaling on ambulatory performance, spatial memory, and receptor dynamics without the need for intranasal insulin delivery, we expressed a constitutively active insulin receptor (IRβ) in the hippocampus of Fischer 344 rats. In aged animals, IRβ was associated with improved memory performance, higher receptor levels, and increased downstream signaling. This work demonstrates the efficacy of molecularly enhancing receptor activity in vivo.

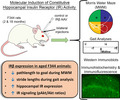

## 1 INTRODUCTION

Insulin and insulin receptor (IR) signaling is known to be an integral component of healthy brain function, and numerous cell types, including endothelial cells, astrocytes, and neurons, express IRs throughout the brain (Zhang et al., 2014). This is particularly important with regard to learning and memory processes. Indeed, the hippocampus, an area of the brain integral for these functions, has consistently shown robust IR expression (Dore, Kar, Rowe, & Quirion, 1997; Unger, Livingston, & Moss, 1991). In hippocampal neurons, these receptors are often localized to postsynaptic densities (particularly in field CA1) where they have been reported to modulate AMPA and NMDA receptors, improve synaptic plasticity, increase hippocampal metabolism, and activate genes and pathways involved in both long‐ and short‐term memory encoding (Adzovic & Domenici, 2014; Pearson‐Leary, Jahagirdar, Sage, & McNay, 2018; Zhao, Chen, Quon, & Alkon, 2004). Additionally, learning has been shown to have a direct impact on hippocampal IR activity, with multiple studies reporting that rats had elevated receptor density and markers of IR signaling after training on the Morris water maze (MWM) behavioral task (Zhao et al., 1999; Zhao et al., 2004).

The connection between insulin and hippocampal learning and memory processes is further supported by evidence of impaired IR signaling in aging and Alzheimer's disease (AD), two phenotypes that are strongly associated with cognitive decline. Specifically, both clinical and preclinical data have shown that aging and AD present with decreased central nervous system (CNS) insulin activity, lower IR expression, and reduced transport of insulin across the blood–brain barrier (Biessels, van der Heide, Kamal, Bleys, & Gispen, 2002; Cholerton, Baker, & Craft, 2011; Frazier, Ghoweri, Anderson, et al., 2019; Rhea & Banks, 2019). Recently, investigators have begun to explore the efficacy of enhancing CNS IR activity via administration of the ligand to combat these impairments. Of the numerous methods that have been investigated, such as intracerebroventricular (ICV) delivery (Grillo, Piroli, Hendry, & Reagan, 2009; Park, Seeley, Craft, & Woods, 2000), the technique of administering insulin via the nasal cavity appears to be the most promising, as it is relatively noninvasive, safe, and easier to perform than other techniques (Frazier, Ghoweri, Anderson, et al., 2019; Freiherr et al., 2013; Hanson & Frey, 2008; Lochhead & Thorne, 2012). Numerous animal studies have shown that intranasal insulin (INI) is associated not only with increased IR signaling markers in the hippocampus (Anderson et al., 2017; Barone et al., 2018; Chen et al., 2014; Frazier, Ghoweri, Sudkamp, et al., 2019; Maimaiti et al., 2016; Rajasekar, Nath, Hanif, & Shukla, 2017), but also with elevated expression of synaptic proteins (Chen et al., 2014), reduced oxidative stress (Barone et al., 2018) and neuroinflammation (Beirami, Oryan, Seyedhosseini Tamijani, Ahmadiani, & Dargahi, 2017; Mamik et al., 2016; Rajasekar et al., 2017), diminished levels of AD markers (e.g., Aβ and hyperphosphorylated tau) (Barone et al., 2018; Chen et al., 2014; Rajasekar et al., 2017), elevated CNS glucose metabolism (Brabazon et al., 2017), and improved memory, learning, and recall (Barone et al., 2018; Brabazon et al., 2017; Maimaiti et al., 2016; Salameh et al., 2015). These results have been corroborated by numerous clinical studies which have also reported positive effects of INI administration on learning, verbal memory performance, and functional abilities (ADCS ADL scores) in older AD and MCI patients (Freiherr et al., 2013). However, while it is clear that insulin is closely tied to learning and memory and that enhancing IR activity can help ameliorate age‐ and AD‐associated cognitive impairments, the precise pathways underlying these effects are still unclear. For this reason, more mechanistic investigations into CNS insulin actions in animal models using molecular methods are needed.

Studies focused on molecular modification of IR activity without involvement of the ligand have recently been employed to study the impact of receptor inhibition or loss of function on learning and memory. Some of the molecular techniques that are commonly used to accomplish this include genetic knockout of IR or associated signaling molecules (Bruning et al., 2000; Cai et al., 2018; Costello et al., 2012; Garcia‐Caceres et al., 2016), the lentiviral delivery of an IR antisense sequence (LV‐IRAS) (Grillo et al., 2015), and the introduction of IR inhibitory peptides (Luckett, Frielle, Wolfgang, & Stocker, 2013; Maimaiti et al., 2017; Paranjape et al., 2010). Results of these studies thoroughly support the importance of IR signaling in cognitive processes, with one showing that CNS‐specific deletion of insulin receptor substrate 2 (IRS‐2), an adaptor protein involved in downstream IR signaling, is associated with the loss of hippocampal metaplasticity in mice (Costello et al., 2012). Specifically, these IRS‐2 knockout mice had impaired long‐term potentiation, reduced hippocampal glutamatergic transmission at CA1 synapses, and decreased levels of IR signaling molecules such as GSK‐3 and pAkt. Interestingly, the deleterious effects of molecular IR inhibition do not appear to be limited to only neurons, as one study has also shown that astrocyte‐specific deletion of IR in mice is associated with reduced astrocytic exocytosis of ATP, decreased purinergic signaling of dopaminergic neurons, and elevated anxiety‐ and depressive‐like behaviors (Cai et al., 2018). Similarly, work from a different group, also in mice, showed that postnatal ablation of astrocytic IRs (Cre/lox system), as well as knockout of astrocytic IRs in the hypothalamus, is associated with reduced astrocytic glucose availability, diminished glucose‐induced activation of pro‐opiomelanocortin (POMC) neurons, and impaired responses to peripheral glucose (Garcia‐Caceres et al., 2016).

However, while these studies have provided significant insights into the processes impacted by impaired IR activity in the brain (a phenomenon strongly implicated in the progression of pathological aging and AD), they do not directly report on the potential mechanisms targeted by exogenous insulin. As the use of INI to elevate IR activity is currently being explored as a therapeutic, it is imperative that molecular approaches that induce a *gain* of function of the brain‐specific IR are also investigated. Here, we attempted to address this need by employing a molecular approach to increase, rather than decrease, IR activity using a constitutively active human IR (IRβ). We expressed this modified receptor in the hippocampus of young and aged Fischer 344 (F344) rats to test the impact of elevated insulin signaling on ambulatory gait performance, receptor expression, and spatial learning and memory. We show that the molecular induction of increased IR signaling mildly altered gait performance and improved memory recall in aged animals performing the MWM task. IRβ expression was also associated with elevated downstream IR signaling markers and total IR density in the hippocampus of aged animals, indicating that prolonged receptor activation does not appear to trigger the downregulation of receptor levels or signaling in these animals. Surprisingly, animals that received the IRβ construct also showed increased nuclear staining (DAPI) reflecting a potential beneficial impact on cell health, although not likely through an enhancement in the number of neurons. The results presented here imply that this alternative approach to increase neuronal IR activity in the hippocampus in vivo without the need for the ligand is safe, effective, and able to alleviate memory impairment in aged animals. This work also highlights the importance of using novel molecular techniques to study IR signaling in the context of age‐related cognitive decline.

## 2 RESULTS

### 2.1 Hippocampal expression of the constitutively active IRβ does not impact overall animal health or gait performance

**Figure 1:**
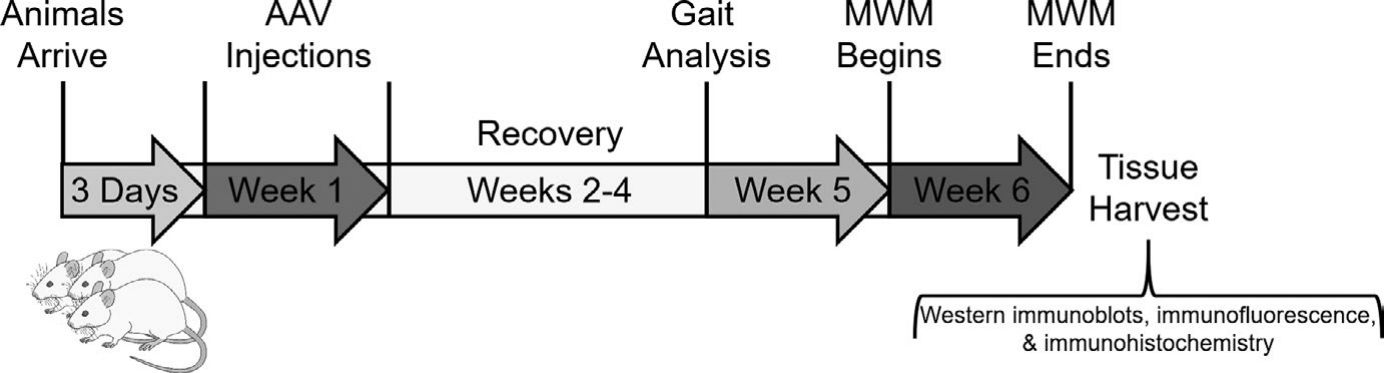
Timeline for AAV injections and behavioral measures performed in young and aged F344 animals. Stereotaxic AAV injections began on Week 1, 3 days after animals arrived. Animals were then given 2–4 weeks of recovery prior to the initiation of gait (Week 5) and MWM (Week 6) measures. Following completion of the MWM, animals were perfused, and tissue was harvested for further analyses

Animals were assessed for changes in body weight following 5 weeks of constitutive insulin signaling driven by the IRβ construct. While a main effect of age was noted (2‐way ANOVA; *F*_(1,29)_ = 247.10, *p* < 0.0001), the AAV treatment did not cause any significant changes in weight in either age‐group (Table 1; *p* > 0.05). Ambulatory performance was measured across age‐ and treatment groups to assess the impact of constitutive hippocampal IR signaling on gait (Figure 1). Although IRβ expression was associated with shorter stride length (Table 1; 2‐way ANOVA; *F*_(1,26)_ = 12.70, *p* = 0.001), it did not appear to impact other measures of gait (*p* > 0.05). Surprisingly, however, a clear aging effect was noted on all measures, with aged animals having fewer crossover mistakes (*F*_(1,26)_ = 9.33, *p* = 0.005), smaller offset differentials (*F*_(1,26)_ = 6.66, *p* = 0.016), and shorter strides (*F*_(1,26)_ = 96.19, *p* < 0.001).

**Table 1:** Measures of animal weight and ambulatory performance in young and aged animals treated with control or IRβ AAVs

	Young		Aged		*p*‐Values	
	Control	IRβ	Control	IRβ	Age	AAV
Weight (g)	282.13 ± 9.33	282.67 ± 12.50	422.56 ± 8.25	432.60 ± 7.62	<0.0001	n.s.
Stride (cm)	1.894 ± 0.038	1.712 ± 0.059	1.447 ± 0.028	1.325 ± 0.048	0.001	0.001
Number of crossovers	0.324 ± 0.058	0.299 ± 0.061	0.122 ± 0.052	0.134 ± 0.062	0.005	n.s.
Offset differentials (cm)	0.170 ± 0.038	0.150 ± 0.025	0.087 ± 0.018	0.090 ± 0.021	0.016	n.s.

While an effect of age on body weight was noted (2‐way ANOVA; *F*_(1,29)_ = 247.10, *p* < 0.0001), no IRβ‐associated changes were present in either age‐group (*p* > 0.05). A robust aging effect was identified on all measures of ambulatory performance: stride (2‐way ANOVA; *F*_(1,26)_ = 96.19, *p* < 0.001), crossovers passing the midline (*F*_(1,26)_ = 9.33, *p* = 0.005), and offset differentials (*F*_(1,26)_ = 6.66, *p* = 0.016). While IRβ expression was associated with shorter stride lengths (*F*_(1,26)_ = 12.70, *p* = 0.001), it did not appear to impact other measures of gait (*p* > 0.05). Non‐significance is indicated by “n.s.” Data represent means ± *SEM*.

### 2.2 Expression of the IRβ receptor is associated with improved MWM performance in aged animals

**Figure 2:**
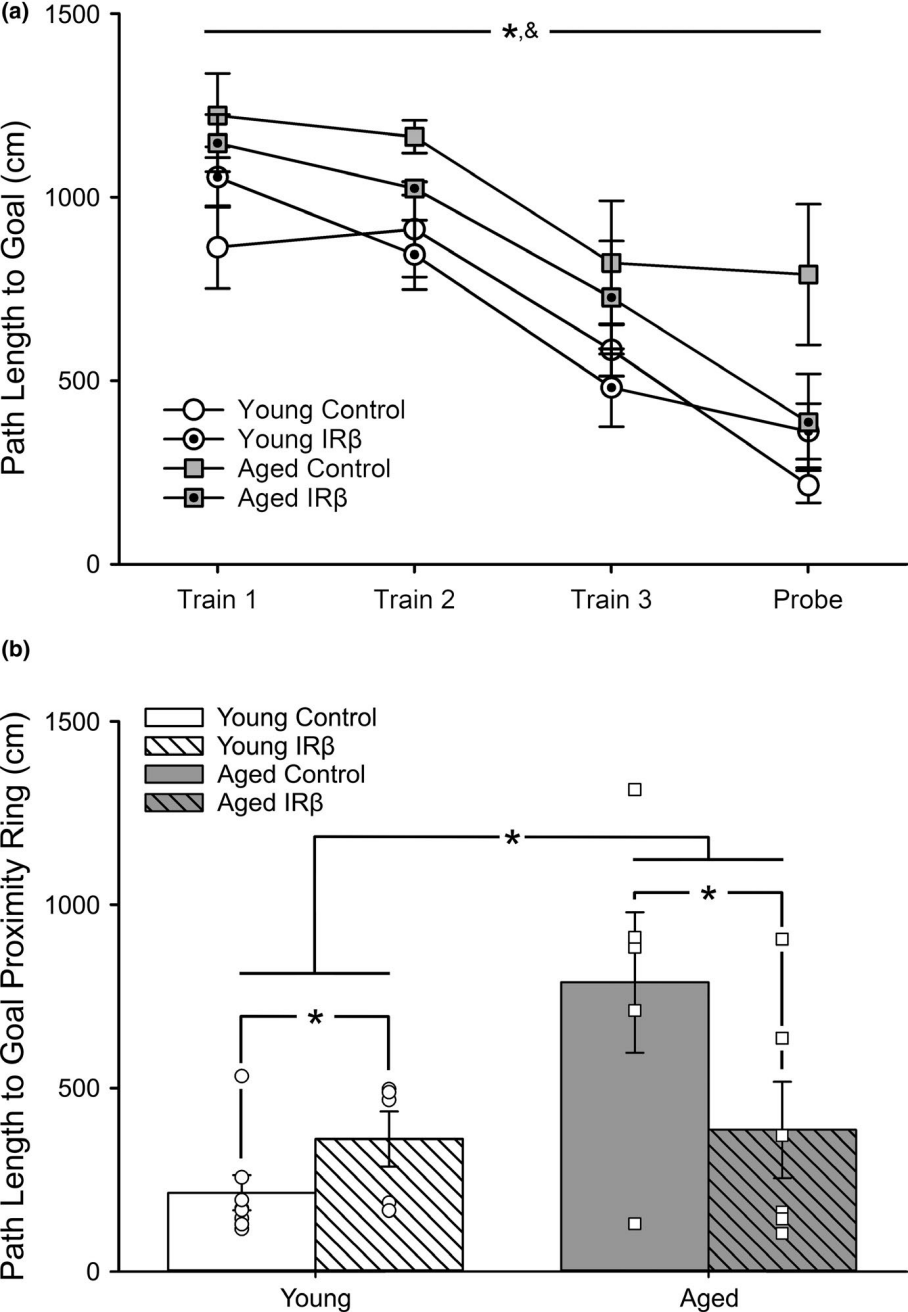
Morris water maze performance in young and aged animals treated with control or IRβ AAVs. (a) Path length to goal across learning (training days) and memory (probe day). All animals (young control *n* = 8, young IRβ *n* = 5, aged control *n* = 5, aged IRβ *n* = 6) learned the task successfully, as indicated by reductions in path length across the training days (3‐way RM ANOVA; *F*_(1.82,36.41)_ = 22.67, *p* < 0.001). As expected, aged animals learned at a slower rate than young animals (3‐way RM ANOVA; *F*_(1,20)_ = 7.97, *p* = 0.012). Asterisk (*) indicates significant main effect of training at *p* < 0.05. Ampersand (&) indicates significant main effect of age at *p* < 0.05. (b) Analysis of memory (24 h) on the probe task revealed both a significant aging effect (2‐way ANOVA; *F*_(1,20)_ = 6.92, *p* = 0.016) and interaction term (2‐way ANOVA; *F*_(1,20)_ = 5.82, *p* = 0.026) whereby aged animals treated with IRβ showed reductions in path length to proximity ring. Asterisks (*) indicate significance at *p* < 0.05. All data represent means ± *SEM*

Animals were assessed for learning and memory performance using the MWM task. All groups showed significant learning across the three training days (Figure 2a; 3‐way RM ANOVA; *F*_(1.82,36.41)_ = 22.67, *p* < 0.001). As expected, aged animals performed more poorly than young, learning at a slower rate (3‐way RM ANOVA; *F*_(1,20)_ = 7.97, *p* = 0.012). Aged animals also had significantly longer path lengths to the goal proximity ring on the memory probe day (Figure 2b; 2‐way ANOVA; *F*_(1,20)_ = 6.92, *p* = 0.016). Interestingly, a significant interaction term was noted on this measure in animals that received the IRβ construct, with older animals displaying evidence of improved memory recall (2‐way ANOVA; *F*_(1,20)_ = 5.82, *p* = 0.026). This suggests that expression of the constitutively active IRβ construct and increases in IR signaling can beneficially impact memory processes in aging.

### 2.3 Aged animals expressing hippocampal IRβ have elevations in downstream IR signaling markers

**Figure 3:**
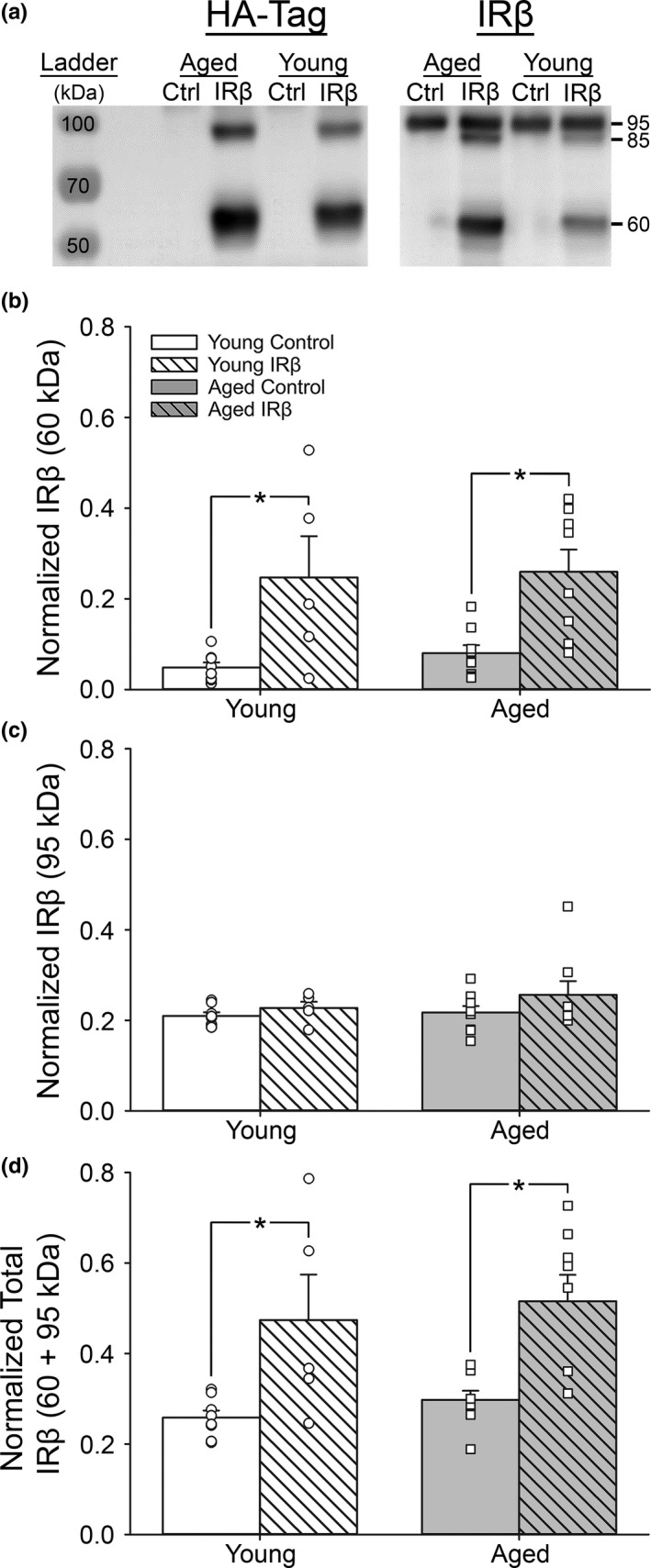
Western immunoblots probing for endogenous IR and truncated IRβ protein in hippocampal tissue from control and IRβ‐treated animals. (a) Representative photomicrograph of Western immunoblots performed on hippocampal tissue taken from 30 animals (young control = 8, young IRβ *n* = 5, aged control *n* = 9, aged IRβ *n* = 8) probing for HA‐tag (left) or the β‐subunit of the IR (right). Expression of the HA‐tag (~60 kDa, left) was used to confirm successful delivery and expression of the IRβ construct (~60 kDa, right). (b) Quantification of ~60 kDa band intensities representing the IRβ construct. IRβ‐treated animals (both young and aged) show a significant increase expression of the modified IRβ receptor compared to controls (2‐way ANOVA; *F*_(1,26)_ = 20.05, *p* < 0.001). (c). Quantification of ~95 kDa band intensities representing the endogenous IR. Endogenous IR did not appear to change across age‐ or treatment groups (*p* > 0.05). (d) Sum of band intensities derived from the ~60 kDa IRβ construct and the ~95 kDa endogenous IR. IRβ‐treated animals (both young and aged) show a significant increase in expression total IRs compared to controls (2‐way ANOVA; *F*_(1,26)_ = 20.13, *p* < 0.001). All data were normalized to total protein levels (Ponceau S staining). Asterisks (*) indicate significance at *p* < 0.05. Data represent means ± *SEM*

Hippocampal homogenates were probed for the HA‐tag, the β‐subunit of the IR, pAkt, and Akt expression using Western immunoblot techniques. The endogenous IR is predicted to have a molecular weight of ~230 kDa, with an ~95 kDa β‐subunit. Based on the combination of the HA‐tag structure (~100 amino acids) and the structure of the modified IRβ receptor used here (~470 amino acids), the total predicted molecular weight of our construct is ~60 kDa. However, analysis of Western immunoblots using an antibody specific to the IR β‐subunit revealed the presence of three, rather than two, distinct bands: the endogenous IR band at ~95 kDa, the modified IRβ band at ~60 kDa, and an additional band at ~85 kDa (Figure 3a, right). While the ~85 kDa band was unexpected, it appears to align with an additional HA‐tag band at a similar molecular weight (Figure 3a, left). This suggests that the proteins at ~85 kDa are either a by‐product of our IRβ construct or a post‐translational modification (i.e., ubiquitination or glycosylation) of the ~60 kDa IRβ protein to target it for degradation and thus are unlikely to significantly contribute to IR signaling. For this reason, the ~85 kDa band was not quantified.

**Figure 4:**
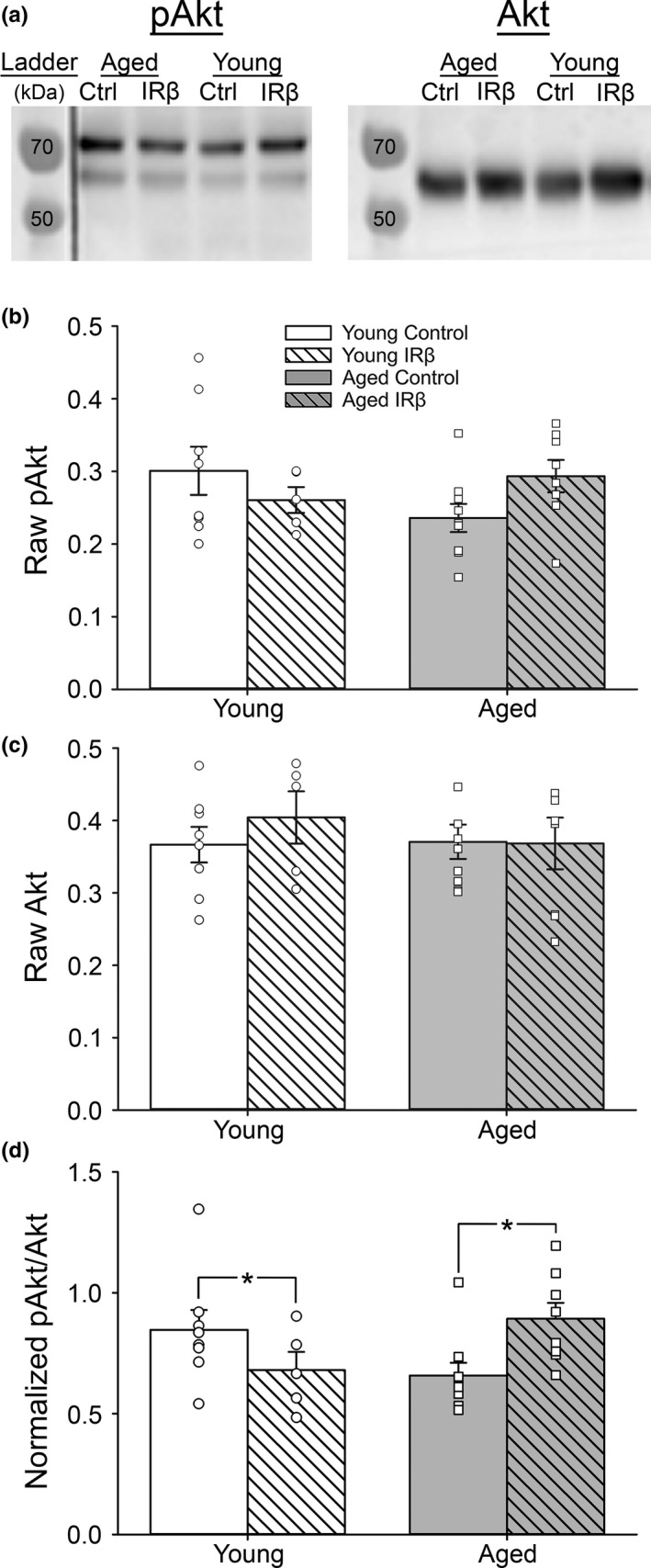
Western immunoblots probing for downstream IR signaling markers in hippocampal tissue from control and IRβ‐treated animals. (a) Representative photomicrograph of Western immunoblots performed on hippocampal tissue taken from 30 animals (young control = 8, young IRβ *n* = 5, aged control *n* = 9, aged IRβ *n* = 8) probing for pAkt (left) or Akt (right). (b‐c) Quantification of pAkt (b) and Akt (c) expression. No differences across age or treatment were detected for either marker (2‐way ANOVA; *p* > 0.05). (d) Quantification of pAkt/Akt ratios indicate that our manipulation was able to impact downstream IR signaling in the hippocampus, as indicated by a significant interaction (2‐way ANOVA; *F*_(1,26)_ = 8.09, *p* = 0.009). Only aged animals responded with increased IR signaling following IRβ treatment. All data were normalized to total protein levels (Ponceau S staining). Asterisks (*) indicate significance at *p* < 0.05. Data represent means ± SEM

In order to assess the level of all functional IRs (both endogenous and the IRβ construct), we measured band intensities from just the ~60 kDa and ~95 kDa bands. As expected, a close parallel between the pattern of HA‐tag expression and expression of the smaller modified receptor was detected (Figure 3a), with control samples having virtually no detectable HA‐tag signal compared to those from IRβ‐treated animals (Figure 3b, 2‐way ANOVA; *F*_(1,26)_ = 20.05, *p* < 0.001). Analysis of ~95 kDa bands alone revealed no significant change between treatment groups (Figure 3c; *p* > 0.05), suggesting that increasing expression of the IRβ construct did not alter endogenous receptor levels. In order to compare the expression of all IRs capable of contributing to downstream signaling, we then combined the intensities of the ~95 kDa and ~60 kDa bands. Analysis indicated that IRβ‐treated animals had significantly higher levels (~2‐fold) of total IR compared to controls (Figure 3d; 2‐way ANOVA; *F*_(1,26)_ = 20.13, *p* < 0.001). To further assess the level of receptor activity, hippocampal homogenates were also probed for pAkt and Akt, two common markers of downstream IR signaling. In aged animals, IRβ treatment was associated with an increase in hippocampal pAkt/Akt ratios compared to controls, while an IRβ‐associated decrease in pAkt/Akt was noted in young animals (Figure 4d; 2‐way ANOVA; *F*_(1,26)_ = 8.09, *p* = 0.009). Neither normalized pAkt nor normalized Akt values differed between age‐ or treatment groups (Figure 4b,c; 2‐way ANOVA; *p* > 0.05). Together, these results suggest that, at least in the F344 animal, the hippocampus does not appear to experience a compensatory reduction in endogenous IR production, even in the presence of elevated signaling.

### 2.4 IRβ expression is associated with increases in immunostained IR‐positive area in the hippocampus

**Figure 5:**
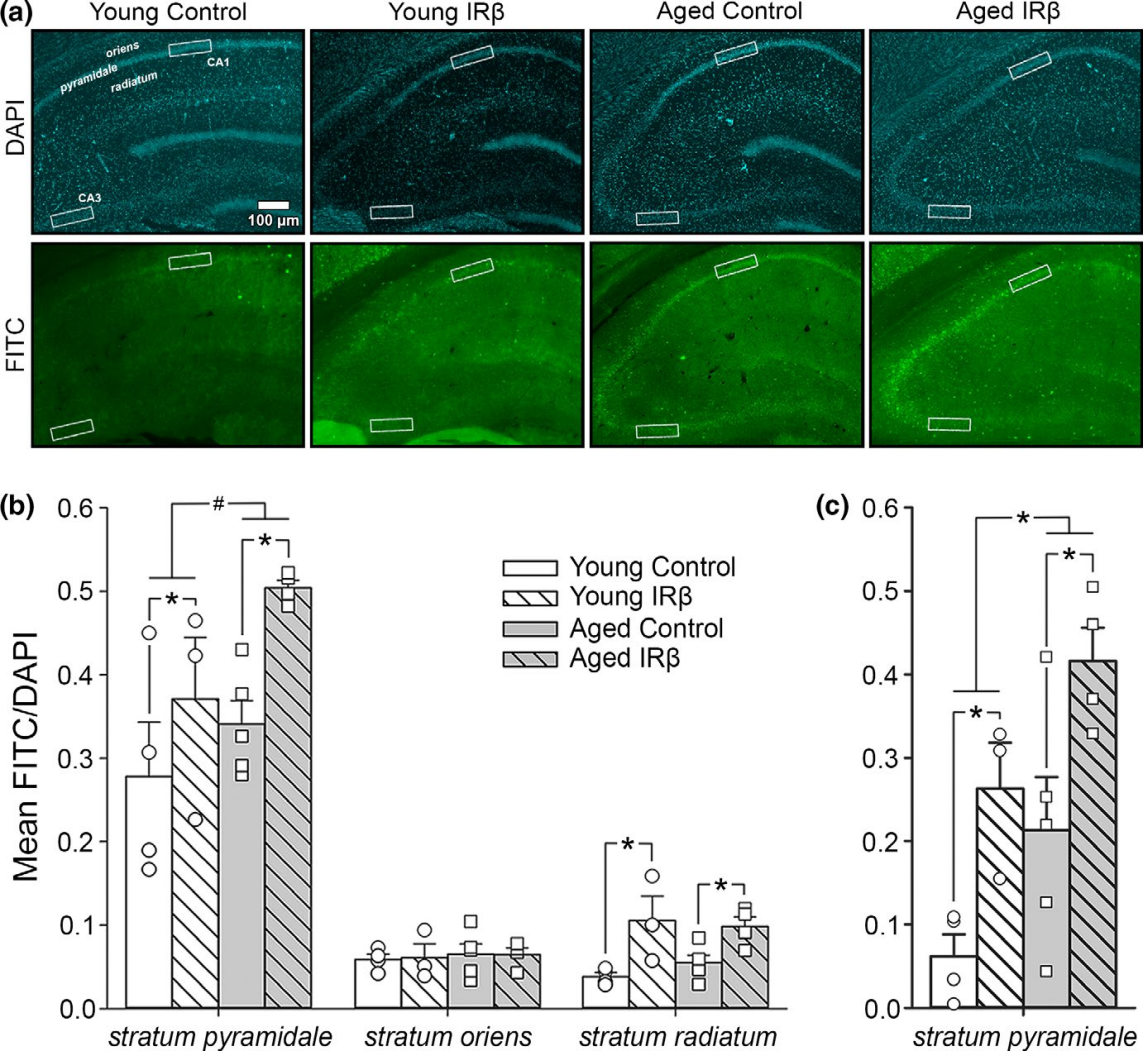
Immunofluorescence of hippocampal tissue sections from control and IRβ‐treated young and aged animals probed for the β‐subunit of the IR. (a) DAPI (blue) and FITC (green) immunofluorescence was used to quantify signal intensity in a ROI (white boxes) across fields CA1 (*strata oriens*,* pyramidale*,* and radiatum*) and CA3 (*stratum pyramidale*) of hippocampal sections from 16 animals (young control *n* = 4, young IRβ *n* = 3, aged control *n* = 5, aged IRβ *n* = 4). (b) Quantification of the IR β‐subunit signal intensity (FITC) normalized to neuronal density (DAPI) in field CA1. Significant increases in normalized signal intensity were detected in IRβ‐treated animals in subfields *stratum pyramidale* (2‐way ANOVA; *F*_(1,12)_ = 7.66, *p* = 0.017) and *stratum radiatum* (*F*_(1,12)_ = 16.45, *p* = 0.002), but not *stratum oriens* (*p* > 0.05). A trend for greater signal intensity in aged animals was also noted in subfield *stratum pyramidale* only (*F*_(1,12)_ = 4.47, *p* = 0.056). (c) Quantification of normalized immunopositive IR β‐subunit signal intensity in *stratum pyramidale* of field CA3. A significant increase in normalized signal was detected in IRβ‐treated animals compared to controls (2‐way ANOVA; *F*_(1,12)_ = 15.01, *p* = 0.002). An aging effect was also noted, with aged animals having greater immunopositive signal than young (*F*_(1,12)_ = 8.48, *p* = 0.013). Asterisks (*) indicate significance at *p* < 0.05. Hash (#) indicates a trend at *p* < 0.10. Data represent means ± *SEM*

To investigate the impact of the IRβ construct on total IR expression, we measured the immunopositive area for the β‐subunit of the IR (endogenous and exogenous) in hippocampal sections. We initially focused on modifying IR expression in primary projecting neurons of the hippocampus (*stratum pyramidale* of field CA1), but also included results measured in field CA3 to investigate potential changes across the dorsal‐ventral axis of the hippocampus. In order to account for the impact of cell number when quantifying immunopositive signals, we normalized the thresholded % area covered of all FITC signals (green) measured in fields CA1 and CA3 to the thresholded % area covered of DAPI (a marker of adenine–thymine‐rich regions of DNA that labels nuclei; blue) measured in *stratum pyramidale* of the corresponding field. We show that IRβ expression was associated with a significant increase in immunopositive area in CA1 subfields *stratum pyramidale* and *stratum radiatum* (Figure 5b; 2‐way ANOVA; *F*_(1,12)_ = 7.66, *p* = 0.017 and *F*_(1,12)_ = 16.45, *p* = 0.002, respectively), but not in *stratum oriens* (*p* > 0.05). A trend for an effect of aging in CA1 *stratum pyramidale* was also noted, with aged animals having greater immunopositive area than that seen in young (2‐way ANOVA; *F*_(1,12)_ = 4.47, *p* = 0.056). Similarly, in *stratum pyramidale* of field CA3, both IRβ and age were associated with elevated immunopositive signals (Figure 5c; 2‐way ANOVA; *F*_(1,12)_ = 15.01, *p* = 0.002 and *F*_(1,12)_ = 8.48, *p* = 0.013, respectively). Analysis of immunopositive DAPI (mean gray value) in fields CA1 and CA3 revealed that IRβ expression was associated with elevated signal intensity across all fields and subfields tested (Table 2). Of note, DAPI signals measured in CA1 *stratum pyramidale* were nearly 2‐fold higher in tissue from IRβ‐treated animals compared to tissue from controls (2‐way ANOVA; *F*_(1,12)_ = 11.92, *p* = 0.005). Surprisingly, these results suggest that elevated insulin signaling in the hippocampus may improve cellular health or growth (albeit not through increasing neuron numbers; see *NeuN immunohistochemistry* below), and also clearly show that our molecular approach was able to alter receptor dynamics.

**Table 2:** Immunopositive DAPI signals in fields CA1 and CA3 of hippocampal sections from young and aged animals treated with control or IRβ AAVs.

DAPI (mean gray value)							
		Young		Aged		*p*‐Values	
		Control	IRβ	Control	IRβ	Age	AAV
CA1	*stratum pyramidale*	99.06 ± 16.32	153.07 ± 3.90	115.03 ± 14.29	149.08 ± 3.93	n.s.	0.005
	*stratum oriens*	75.31 ± 12.65	113.89 ± 1.58	88.26 ± 11.79	116.83 ± 4.05	n.s.	0.007
	*stratum radiatum*	84.39 ± 13.66	129.82 ± 4.34	99.13 ± 11.77	132.78 ± 4.23	n.s.	0.003
CA3	*stratum pyramidale*	83.94 ± 13.45	127.16 ± 2.81	98.82 ± 12.29	131.63 ± 5.05	n.s.	0.005

A robust effect of IRβ treatment on field CA1 immunopositive DAPI signal across the three subfields measured was detected in both young and aged animals: *stratum pyramidale* (*F*_(1,12)_ = 11.92, *p* = 0.005), *stratum oriens* (*F*_(1,12)_ = 10.66, *p* = 0.007), and *stratum radiatum* (*F*_(1,12)_ = 13.6, *p* = 0.003). Similarly, IRβ was also associated with increased DAPI signals in subfield *stratum pyramidale* of field CA3 (*F*_(1,12)_ = 12.11, *p* = 0.005). No effect of age was noted in either of the fields measured (*p* > 0.05). Non‐significance is indicated by “n.s.” Data represent means ± *SEM*.

As our AAV treatment was targeted to CA1, the presence of immunopositive signal in *stratum pyramidale* of field CA3 could be attributed to neuronal terminals incorporating the virus, leading to expression in the somatic region of this field. However, we did note significantly smaller levels of immunopositive signal in CA3 compared to CA1 (3‐way ANOVA; *F*_(1,24)_ = 15.04, *p* = 0.001), which is perhaps not surprising given our intent to target field CA1. Interestingly, we also report significant main effects of age (3‐way ANOVA; *F*_(1,24)_ = 12.82, *p* = 0.002) and IRβ treatment (*F*_(1,24)_ = 22.41, *p* < 0.0001) in both fields.

### 2.5 NeuN immunohistochemistry of hippocampal sections

To investigate the impact of IRβ expression and increased IR signaling on neuronal density or the potential infiltration and expansion of other cell types (i.e., astrocytes or microglia), we quantified NeuN‐positive neurons in subfield *stratum pyramidale* of hippocampal fields CA1 and CA3. Quantification of field CA3 showed a significant effect of age (Table 3; 2‐way ANOVA; *F*_(1,12)_ = 18.90, *p* = 0.001), as well as a trend for a reduction in neuron number in IRβ‐treated animals compared to controls (*F*_(1,12)_ = 4.17, *p* = 0.064). Surprisingly, this was not reflected in field CA1, as no effect of age or IRβ was detected (*p* > 0.05, respectively), suggesting that the IRβ‐associated increase in immunopositive DAPI signals reported in Table 2 may be mediated through mechanisms independent of neurogenesis.

**Table 3:** Number of neurons in fields CA1 and CA3 of hippocampal sections following NeuN immunohistochemistry.

Number of NeuN‐positive neurons per 100 µm							
		Young		Aged		*p*‐Values	
		Control	IRβ	Control	IRβ	Age	AAV
CA1	*stratum pyramidale*	43.88 ± 4.32	42.78 ± 2.27	42.00 ± 3.34	33.42 ± 1.16	n.s.	n.s.
CA3	*stratum pyramidale*	35.50 ± 1.85	30.72 ± 1.03	27.23 ± 0.74	25.83 ± 2.00	0.001	n.s.^#^

No effect of IRβ treatment or age was detected on neuron numbers in *stratum pyramidale* of field CA1 following NeuN immunohistochemistry of hippocampal sections (*p* > 0.05). In field CA3, a significant age‐associated decrease in the number of NeuN‐positive neurons was noted (2‐way ANOVA; *F*_(1,12)_ = 18.90, *p* = 0.001), as well as a trend for reduced neuron numbers in IRβ‐treated animals compared to controls (*F*_(1,12)_ = 4.17, *p* = 0.064). Non‐significance is indicated by “n.s.” A superscript hash (^#^) indicates a trend at *p* < 0.10. Data represent means ± *SEM*.

## 3 DISCUSSION

In addition to its role in spatial learning and memory, the hippocampus is also associated with the processing of information necessary for controlled ambulatory performance. In fact, age‐associated changes in the control of movement are well recognized in the clinic (Beauchet, Allali, Launay, Herrmann, & Annweiler, 2013), where greater variability in these measures is correlated with structural or functional differences not only in the somatosensory cortex, but also in the hippocampus, anterior cingulate gyrus, and basal ganglia (Tian et al., 2017). For this reason, we sought to explore whether elevating hippocampal insulin signaling in young and aged animals through the use of a neuron‐specific molecular approach that does not require administration of the ligand (Frazier et al., 2018, 2020; Frazier, Ghoweri, Anderson, et al., 2019; Lebwohl, Nunez, Chan, & Rosen, 1991) is a viable method to improve memory processes or ambulatory performance. We also attempted to corroborate our previous work using INI delivery in the same animal model (F344 rats) at comparable ages (Anderson et al., 2017; Frazier, Ghoweri, Sudkamp, et al., 2019; Maimaiti et al., 2016). Unlike INI, which bypasses the blood–brain barrier and is able to impact numerous brain regions and cell types (Chen et al., 2014; Lochhead & Thorne, 2012), the molecular technique employed here was designed to increase insulin signaling in hippocampal neurons only through the use of the synapsin I promoter, which has a high specificity for neurons (McLean et al., 2014). This distinction is important, considering that while highly expressed in neurons, the IR is also found in other cells such as astrocytes (Cai et al., 2018; Garcia‐Caceres et al., 2016), making it difficult to elucidate the exact mechanism of INI action in the brain. To address this, we provide a method that is inherently more precise and directs IR elevations to fewer cell types, allowing for a more targeted investigation of hippocampal insulin activity. Similar to our previous results using INI lispro or detemir (Maimaiti et al., 2016), the 5‐week regimen (Figure 1) of elevated IR signaling used here was able to mitigate the age‐dependent decline in memory (Figure 2), as seen by reduced path lengths to goal in the aged IRβ‐treated animals. While the treatment did significantly reduce stride length by approximately 10% in both age‐groups, it is not clear what the importance of this result is, given that other measures of ambulatory performance were not altered (Table 1). However, this result does highlight the importance of the hippocampus in controlling at least some aspects of gait (Tian et al., 2017) and points to potential insulin‐mediated reductions in the afterhyperpolarization (Maimaiti et al., 2016; Pancani et al., 2013) which could perhaps alter network activity in the microcircuitry guiding motor control. Additionally, reductions in the number of midline crossovers, stride length, and offset differentials in the aged group were noted (Table 1). This may reflect greater inquisitive behavior in the younger animals, or perhaps an underestimation of mobility (e.g., wider haunches) of larger animals in a relatively confined space. Interestingly, these results were corroborated by similar reductions in offset differentials from midline in the aged group, likely due to more focused and determined ambulatory behavior in these animals. Clearly, more investigations into the role of hippocampal IR activity in the control of gait are warranted.

The potential mechanisms by which IRβ expression improved spatial memory may have been driven by several factors. One consideration could be the enhancement of insulin signaling beyond physiological levels, as seen by increased hippocampal IR density (Figure 3) and downstream IR signaling molecules (Figure 4). This is further supported by increased immunopositive signal in hippocampal fields CA1 (*stratum pyramidale* and *stratum radiatum*) and CA3 (*stratum pyramidale*) in IRβ‐treated animals compared to controls (Figure 5b,c). These results could imply a redressing of an insulin starvation state, as studies have shown that the aged brain has reduced IR expression, diminished insulin transport into the CNS, and may even experience insulin resistance (Biessels et al., 2002; Cholerton et al., 2011; Frazier, Ghoweri, Anderson, et al., 2019; Rhea & Banks, 2019). However, we have not observed these changes in the F344 animal model of aging; in fact, prior publications from our group have found neither age‐associated reductions in pAkt/Akt ratios (Anderson et al., 2017) nor reduced I^125^ insulin binding (Frazier, Ghoweri, Sudkamp, et al., 2019) in the hippocampus or other areas of the brain. Further, another study using a different antibody for the β‐subunit of the IR in this same animal model at later ages (17–18 months) also showed no aging differences in IR expression (Pancani et al., 2013). Discrepancies between our findings and those of others could be attributed to multiple factors, including the study type performed (clinical vs. preclinical), the specific ages of the subjects tested, or the particular strain of the animal model used (i.e., Sprague‐Dawley vs. F344 rats). Additionally, investigations of aging processes in healthy, older subjects (18–21 months) may avoid confounds associated with use of animals closer to the end of their lifespan (~23–31 months). Thus, it could be that F344 rats do not experience a reduction in IR expression with age but still benefit from a supraoptimal enhancement of insulin signaling through yet unknown mechanisms.

In addition to enhancing downstream IR signaling, the positive impact of elevating IRβ expression on spatial learning and memory could also be attributed to insulin's ability to reduce neuroinflammation (Beirami et al., 2017; Freiherr et al., 2013; Mamik et al., 2016; Rajasekar et al., 2017) and work as a neuroprotective agent (Calvo‐Ochoa & Arias, 2015). In fact, we report IRβ‐associated increases in DAPI immunofluorescence (Table 2), suggesting greater cell numbers in these regions. In order to further investigate the impact of IRβ expression on neuronal density or potential infiltration/expansion of other cell types (i.e., astrocytes or microglia), we quantified NeuN‐positive neurons in fields CA1 and CA3. Surprisingly, counts of NeuN‐positive neurons did not mirror the reported IRβ‐associated increase in DAPI signal in either field. This suggests that further investigations on the impact of increased insulin signaling in astrocytes or microglia are warranted, as these cell types are known to express the IR (Zhang et al., 2014) and also participate in spatial learning and memory processes (Sompol et al., 2017).

Other mechanisms that could potentially underly the impact of IRβ on spatial learning and memory reported here include enhanced glucose metabolism and/or expression of glucose transporters (Frazier et al., 2020; Grillo et al., 2009; Pearson‐Leary et al., 2018; Uemura & Greenlee, 2006) or reductions in neuronal calcium dysregulation (Frazier et al., 2017; Maimaiti et al., 2016, 2017; Pancani et al., 2013; Stella, Bryson, & Thoreson, 2001; Thibault et al., 2013), as these processes have a rich history of association with mechanisms tied to cognitive decline in aging or AD. Additionally, while the AAV delivery initially targeted field CA1, we did note alterations in IR density in field CA3 (Figure 5). However, neuronal communication in field CA3 neurons is often associated with short‐term spatial working memory (Duncan, Tompary, & Davachi, 2014; Lee & Kesner, 2003) and thus is not likely to have participated in the enhancement of long‐term memory recall during the more delayed 24‐h probe trial. It is also interesting to note that only two subfields (*strata pyramidale* and *radiatum*) of CA1 responded to the IRβ treatment used here. One potential explanation for a lack of effect in *stratum oriens* is the presence of numerous interneurons or basket cells in this region, as these cells are important for trace encoding and fear conditioning‐type behaviors (Lovett‐Barron et al., 2014) and are less likely to be targeted by our construct. Further, *strata pyramidale* and *radiatum* form the last synaptic structures within the hippocampal circuit and are therefore more likely to impact spatial navigation and performance (i.e., the MWM task). Finally, the separation of these subfields by mere microns suggests that the IRβ construct did not have a generalized effect throughout the hippocampus; nevertheless, future studies regarding the impact of insulin signaling between hippocampal fields and subfields are still warranted.

Although the findings reported here indicate that our molecular approach was safe, as highlighted by the lack of noticeable ill effects on the overall health of the animals (Table 1), the possibility of aberrant cellular growth caused by sustained signaling of a growth hormone must be considered. However, given that only a small IRβ‐associated increase in hippocampal DAPI signals was detected (Table 2) along with no significant IRβ‐associated changes in NeuN‐positive neurons (Table 3), it is unlikely that our treatment led to uncontrolled growth. Interestingly, our results also indicated that neither endogenous IR expression nor downstream IR signaling was reduced following several weeks of sustained insulin activity driven by the modified IRβ receptor (Figures 3 and 4). While the lack of endogenous IR downregulation is surprising, it could be attributed to functional differences between peripheral and CNS receptor isoforms. In fact, some work has shown that the brain‐specific IR isoform (IR‐A) does not downregulate as quickly as the peripheral IR‐B isoform following prolonged incubation with the ligand (Boyd & Raizada, 1983). Additionally, while our results show that the constitutively active IRβ construct had very little impact on gait in the current study, this treatment did not critically impair ambulatory performance either, further supporting the overall safety of this technique.

In summary, the work presented here indicates that our molecular approach for increasing neuronal IR activity in the hippocampus in vivo without the need for the ligand is well‐tolerated, effective, and able to alleviate age‐dependent spatial memory impairments on the MWM task. Importantly, our findings also highlight the value of exploring novel molecular techniques to assess the impact of elevated insulin signaling in the CNS and directly study the specific mechanisms and pathways associated with IR activation in the hippocampus across different cell types.

## 4 EXPERIMENTAL PROCEDURES

### 4.1 AAV construction

The AAV donor plasmids (OTHp180710A1 and OTHp180710A2) were constructed by the University of Kentucky Genetic Technologies Core using a modified pAAV‐CaMKIIa‐hM4D(Gi)‐mCherry backbone (gift from Bryan Roth; plasmid #50477, Addgene, Watertown, MA). To limit expression of the AAVs to neurons, the neuron‐specific human synapsin I promoter was ligated into this backbone to replace the original CaMKIIa promoter. An AAV9 viral coat protein was chosen for both constructs (control and IRβ), as it has been shown to have enhanced transduction compared to other serotypes (McLean et al., 2014). AAV9‐coated viruses were produced by co‐transfecting 293LTV cells with the donor plasmid, pAAV2/9 (U Penn Vector Core), and pAdΔF6 (gift from James M. Wilson; plasmid #112867, Addgene). The viruses were purified by ultracentrifugation through an iodixanol step gradient and then concentrated by washing with PBS on an Amicon 15 (100,000 MWCO) spin filter. AAV titers were as follows: control 3.47E+13 genomes/mL, IRβ 4.46E+13 genomes/mL. The final viral preparations were then frozen at −80°C until needed.

### 4.2 Animal models and stereotaxic AAV delivery

Male F344 rats, aged 2 (*n* = 14) or 18 months (*n* = 19), were obtained from the National Institute on Aging colony. Animals were housed in pairs, tail marked for identification, maintained on a 12 h ON/12 h OFF light schedule, and fed Teklad global 18% protein rodent diet (2018; Harlan Laboratories, Madison, WI) ad libitum. For the next week, beginning on the fourth day after arrival, all animals received injections (2 µl per side, 0.2 µl/min) of either a control AAV containing a neuron‐specific synapsin promoter and a fluorescent marker (mCherry) or an experimental AAV containing the synapsin promoter, mCherry, and the constitutively active IRβ receptor (Lebwohl et al., 1991) in the CA1 region of the hippocampus (young control *n* = 8, young IRβ *n* = 6, aged control *n* = 9, aged IRβ *n* = 10). Stereotaxically guided AAV delivery was accomplished on a KOPF frame (David Kopf Instruments; Tujunga, CA) equipped with a Neurostar robotic device (v4.6.1; Tübingen, Germany). The coordinates used were AP: −4 mm, ML: ±2.2 mm, and DV: −1.8 mm. After injections, the animals were allowed to heal and express the construct for 4 weeks. Experimenters were blinded to the treatment groups and codes were only revealed after statistical analyses were performed.

Beginning on the fifth week post‐surgery, all animals underwent 2 days (Monday and Tuesday) of gait behavioral analysis followed by a 2 day rest (Figure 1). On Friday of week five, animals began the MWM test. One aged IRβ animal was unable to complete the MWM task due to extreme lethargy. Upon completion of the MWM test, animals were sacrificed for tissue analysis. At the time of sacrifice, no differences in animal weights were noted between groups (in g: young control 282.1 ± 9.3, young IRβ 282.7 ± 12.5, aged control 422.6 ± 8.2, aged IRβ 432.6 ± 7.6; 2‐way ANOVA, *p* = 0.6). The central portions of hippocampi from 15 animals (7 young and 8 aged) were removed and reserved for use in a separate study. For these animals, only the hippocampal extremities were frozen for use in the Western immunoblot measures presented here. The remaining 18 animals (7 young and 10 aged) underwent saline perfusion (ice‐cold 0.9% saline in dH_2_O, 10 min). The entire left hemispheres of saline‐perfused brains were post‐fixed (4% PFA and 0.05% picric acid in 1X PBS at 4°C for ~48 h, 15% sucrose in 1X PBS at 4°C for ~24 h, then stored in antifreeze [15% sucrose and 30% ethylene glycol in 1X PBS] at −20°C) for immunofluorescence, while the hippocampi from right hemispheres were flash‐frozen on dry ice for Western immunoblots.

### 4.3 Gait behavioral tests

Animals were assessed for gait measures over 2 days on the fifth week post‐surgery. Each animal was given one untracked test run to acclimate to the task, which consisted of a single, continuous, even‐speed walk down a corridor ending at their home cage. The corridor was then lined with paper and the animal's fore or hind paws were coated in nontoxic black tempera paint (Crayola LLC, Easton, PA). Animal tracks were recorded once for fore paw positions and once for hind paw positions on each of the two testing days. Tracks were measured for stride, number of crossovers passing the midline, and offset differentials (defined as the sum of the absolute differences between the left and right fore paws' offsets from the midline). Stride data were derived from an average of at least seven steps of both fore and hind paws across the two testing days, while measures of crossovers and offset differentials were derived from fore paws only. A number of crossovers passing the midline were then normalized to step number for each animal. For stride and offset differentials, data were normalized to the average width of haunches based on 6 young and 6 aged animals (in cm: young 7.37 ± 0.14, aged 8.74 ± 0.8). We present gait results from 30 animals (young control *n* = 8, young IRβ *n* = 5, aged control *n* = 9, aged IRβ *n* = 8).

### 4.4 MWM behavioral tests

Spatial learning and memory were tested using the MWM. The diameter of the testing pool was 190 cm. Water temperature was maintained between 25 and 26°C. Black tempera paint (Crayola) was used to make the water opaque. A 15 cm escape platform was placed 1.5 cm below the water's surface. Each animal was trained with 3 trials/day, with an inter‐trial interval of 150 s. For each trial, animals were placed in the pool and allowed 60 s to find the platform. A semi‐random drop location was used for each trial. Animals were required to remain on the platform for 30 s before being moved to a heated holding chamber for ~2 min. Prior to training, a visual acuity test was performed using a white cup placed above the platform for three consecutive trials (day 0; fifth week post‐surgery). Beginning on the sixth week post‐surgery, animals underwent three training days (3 trials/day; days 1‐3), then a probe day with the platform removed (1 trial/day; day 4). An Ethovision acquisition and analysis software (v14, Noldus Information Technology, Wageningen, Netherlands) was used to track and measure animal movement. For probe day data analysis, pathlength measures were determined from the start of the trial until the animal reached a proximity ring ~5 cm around the platform. Of the 32 animals that underwent MWM testing, a total of 8 were removed prior to statistical analysis. Five of these animals (2 aged control, 3 aged IRβ) were removed based on a visual acuity filter (failing to find the platform during all three trials on both cue day and at least one training day), while two (1 young IRβ, 1 aged IRβ) were removed based on our Western immunoblot HA‐tag filter (having no detectable expression of the IRβ AAV HA‐tag; see *Western immunoblots* below). The eighth animal (1 aged IRβ) was removed based on both our visual acuity filter and the HA‐tag filter. We present MWM performance measures from 24 animals (young control *n* = 8, young IRβ *n* = 5, aged control *n* = 5, aged IRβ *n* = 6).

### 4.5 Western immunoblots

All Western immunoblots were conducted using 8% fresh‐cast gels with overnight wet transfer at 15°C and overnight primary antibody incubation at 15°C. For HA‐tag immunoblots, membranes were placed in 5% BSA in TBST for both blocking and antibody incubations. For pAkt, Akt, and total IR β‐subunit immunoblots, membranes were blocked in 8% nonfat dry milk in TBST, while antibodies were diluted in 1% nonfat dry milk in TBST. The same protein concentration (50 µg) was loaded for each sample. Membranes were developed using chemiluminescence (ECL Plus, Thermo Fisher Scientific, Waltham, MA) and digitally imaged on a Syngene G:Box (Syngene International LTD, Bangalore, India). Bands were quantified using ImageJ and normalized to total protein load (Ponceau S). Bands obtained from two separate membranes were then averaged within treatment groups. To determine whether successful IRβ expression was obtained, HA‐tag (HA‐Tag C29F4, #3724, Cell Signaling Technologies, Danvers, MA; 1:2000) was first measured in tissue samples from all 33 animals in the study. Of the 16 animals that received the experimental IRβ AAV, only 3 (1 young and 2 aged) did not have detectable HA‐tag expression. Samples from these animals were not included in subsequent immunoblot measures. The remaining samples were then probed for pAkt (Ser473, #4051, Cell Signaling Technologies; 1:1000), Akt (#4685, Cell Signaling Technologies; 1:1000), and total IR β‐subunit (#ab69508, Abcam, Cambridge, United Kingdom; 1:1000) expression. We present Western immunoblot measures of tissues samples taken from 30 animals (young control = 8, young IRβ *n* = 5, aged control *n* = 9, aged IRβ *n* = 8).

### 4.6 Immunofluorescence of hippocampal sections

Tissue sections (35 µm) were obtained from post‐fixed left hippocampi and probed using a modified Abcam IHC protocol for floating sections. The tissue was blocked in 8% goat serum for 2 h, followed by three nights of incubation (15°C) with primary antibody (IRβ, #ab69508, Abcam; 1:500) and 2 h of incubation (room temperature) with a FITC secondary antibody (Alexa Fluor^®^ 555, #ab150114, Abcam; 1:1000). Tissue sections were mounted on subbed glass slides and cover slipped with ProLong™ Diamond Antifade Mountant with DAPI (#P36962, Thermo Fisher Scientific) for imaging using a spectral analysis software package and camera (Nuance^®^, CRi Inc., Hopkinton, MA) with the ability to scan fluorescence wavelengths in 10 nm increments using long‐pass emission filters (DAPI: >400 nm LP; FITC: >500 nm LP). Excitation filters were centered on the DAPI (350 ± 50 nm) and the FITC (470 ± 40 nm) fluorophores. Settings for image acquisition were the same across all sections imaged. Pseudo‐colored images were converted to grayscale prior to analysis. ImageJ was used to quantify the % area covered of immunopositive DAPI and FITC signals in fields CA1 and CA3 across hippocampal sections (3 sections/subject). The same region of interest (ROI) was used for each tissue section. To account for changes in cell number, FITC signals were normalized to the immunopositive DAPI measured in *stratum pyramidale* of their respective fields (CA1 or CA3), as this subfield is comprised of neuronal soma and thus provides the most accurate representation of neuronal cell density. As an additional measure of cell number, the mean gray value of immunopositive DAPI signal only was also measured in *stratum pyramidale* of both fields. We present immunofluorescence results from a total of 16 animals (young control *n* = 4, young IRβ *n* = 3, aged control *n* = 5, aged IRβ *n* = 4).

### 4.7 NeuN immunohistochemistry of hippocampal sections

Hippocampal tissue sections were probed using an anti‐NeuN primary antibody (#MAB377, EMD Millipore, Burlington, MA; 1:400), a biotinylated secondary antibody (VectaStain Elite ABC‐HRP Kit, #PK‐6102, Vector Laboratories, Burlingame, CA; 1:400), and a free‐floating protocol for sections. Briefly, sections were first incubated in 3% hydrogen peroxide in methanol for 30 min to block endogenous peroxidase and then incubated in a blocking solution (3% bovine serum albumin, 0.5% Triton X‐100, and 0.01 M PBS) for 1 h to reduce background, followed by a third overnight incubation in blocking solution containing primary antibody. The next day, sections were rinsed with PBS and incubated (1 h) with biotinylated secondary antibody in blocking solution, followed by a second incubation (1 h) in a biotin amplification solution, then a final PBS rinse. Sections were developed using a DAB peroxidase (HRP) substrate. Sections were then mounted on glass slides, air‐dried, and coverslipped using VectaMount (#H‐5000‐60; Vector Laboratories). Brightfield images of mounted tissue sections were obtained using a Nuance^®^ camera (CRi Inc.). For quantification of NeuN‐positive neurons, three separate investigators manually counted all positive neurons within a randomly selected ROI (100 µm) in *stratum pyramidale* of fields CA1 and CA3. Investigators were blinded to the treatment groups until statistical analyses were completed. We present NeuN immunohistochemistry results derived from a total of 16 animals (young control *n* = 4, young IRβ *n* = 3, aged control *n* = 5, aged IRβ *n* = 4).

### 4.8 Statistical analysis

All results were derived (GraphPad Prism v8.4, GSL Biotech LLC, Chicago, IL) using either a two‐way analysis of variance (2‐way ANOVA), a three‐way analysis of variance (3‐way ANOVA), or a three‐way analysis of variance with repeated measures (3‐way RM ANOVA). Tukey's post hoc tests were used for all ANOVAs. All data are reported as means ±SEM. Significance for all measures was set at *p* < 0.05.

## CONFLICT OF INTEREST

The authors report no conflicts of interest.

## AUTHOR CONTRIBUTIONS

HNF, KLA, and OT contributed to experimental conception and design; KLA and RLL performed gait experiments and analysis; HNF, KLA, AOG, and RLL performed MWM experiments and analysis; KLA performed Western immunoblots and analysis; KLA and TRH performed immunofluorescence and analysis; PS and KLA performed immunohistochemistry; HNF, GJP, and MDM designed and provided viral constructs; and HNF and OT wrote the manuscript during quarantine. All authors read and approved the submitted manuscript.

## Supporting information

Supplementary Material

## Data Availability

Data are available as supplementary material.
